# The Physicians Visiting List for 1878

**Published:** 1878-10-20

**Authors:** 


The Physicians Visiting List for 1878. Twenty-eighth year of its publication, Philadelphia. Lindsay & Blakiston—Sizes from 25 to 100 patients, and prices varying from $1.00 to $3.00. A work of great usefulness and convenience to the practioner.

